# ProDis-ContSHC: learning protein dissimilarity measures and hierarchical context coherently for protein-protein comparison in protein database retrieval

**DOI:** 10.1186/1471-2105-13-S7-S2

**Published:** 2012-05-08

**Authors:** Jingyan Wang, Xin Gao, Quanquan Wang, Yongping Li

**Affiliations:** 1 King Abdullah University of Science and Technology (KAUST), Mathematical and Computer Sciences and Engineering Division, Thuwal, 23955-6900, Saudi Arabia; 2 Shanghai Institute of Applied Physics, Chinese Academy of Sciences, 2019 Jialuo Road, Jiading District, Shanghai 201800, China; 3 Shanghai Key Laboratory of Intelligent Information Processing, School of Computer Science, Fudan University, Shanghai 200433, China

## Abstract

**Background:**

The need to retrieve or classify protein molecules using structure or sequence-based similarity measures underlies a wide range of biomedical applications. Traditional protein search methods rely on a pairwise dissimilarity/similarity measure for comparing a pair of proteins. This kind of pairwise measures suffer from the limitation of neglecting the distribution of other proteins and thus cannot satisfy the need for high accuracy of the retrieval systems. Recent work in the machine learning community has shown that exploiting the global structure of the database and learning the contextual dissimilarity/similarity measures can improve the retrieval performance significantly. However, most existing contextual dissimilarity/similarity learning algorithms work in an unsupervised manner, which does not utilize the information of the known class labels of proteins in the database.

**Results:**

In this paper, we propose a novel protein-protein dissimilarity learning algorithm, ProDis-ContSHC. ProDis-ContSHC regularizes an existing dissimilarity measure *d_ij _*by considering the contextual information of the proteins. The context of a protein is defined by its neighboring proteins. The basic idea is, for a pair of proteins (*i*, *j*), if their context N(i) and N(j) is similar to each other, the two proteins should also have a high similarity. We implement this idea by regularizing *d_ij _*by a factor learned from the context N(i) and N(j).

Moreover, we divide the context to hierarchial sub-context and get the contextual dissimilarity vector for each protein pair. Using the class label information of the proteins, we select the relevant (a pair of proteins that has the same class labels) and irrelevant (with different labels) protein pairs, and train an SVM model to distinguish between their contextual dissimilarity vectors. The SVM model is further used to learn a supervised regularizing factor. Finally, with the new **S**upervised learned **Dis**similarity measure, we update the **Pro**tein **H**ierarchial **Cont**ext **C**oherently in an iterative algorithm--**ProDis-ContSHC**.

We test the performance of ProDis-ContSHC on two benchmark sets, i.e., the ASTRAL 1.73 database and the FSSP/DALI database. Experimental results demonstrate that plugging our supervised contextual dissimilarity measures into the retrieval systems significantly outperforms the context-free dissimilarity/similarity measures and other unsupervised contextual dissimilarity measures that do not use the class label information.

**Conclusions:**

Using the contextual proteins with their class labels in the database, we can improve the accuracy of the pairwise dissimilarity/similarity measures dramatically for the protein retrieval tasks. In this work, for the first time, we propose the idea of supervised contextual dissimilarity learning, resulting in the ProDis-ContSHC algorithm. Among different contextual dissimilarity learning approaches that can be used to compare a pair of proteins, ProDis-ContSHC provides the highest accuracy. Finally, ProDis-ContSHC compares favorably with other methods reported in the recent literature.

## Background

Proteins are linear chains of amino acids. The polypeptide chains are folded into complicated three-dimensional (3D) structures. With different structures, proteins are able to perform specific functions in biological processes [1-14]. To study the structure-function relationship, biologists have a high demand on protein structure retrieval systems for searching similar sequences or 3D structures [15]. Protein pairwise comparison is one of the main functions of such retrieval systems [16]. The need to retrieve or classify proteins using 3D structure or sequence-based similarity underlies many biomedical applications. In drug discovery, researchers search for proteins that share specific chemical properties as sources for new treatment. In folding simulations, similar intermediate structures might be indicative of a common folding pathway [17].

### Related work

The structural comparison problem in a protein structure retrieval system has been extensively studied. In [18], a rapid protein structure retrieval system named ProtDex2 was proposed by Aung and Tan [18] , in which they adopted the information retrieval techniques to perform rapid database search without accessing to each 3D structure in the database. The retrieval process was based on the inverted-file index constructed on the feature vectors of the relationship between the secondary structure elements (SSEs) of all the protein structures in the database. In order to evaluate the similarity score between a query protein structure and a protein structure in the database, they adopted and modified the well-known ∑(*tf *× *idf*) *scoring scheme *commonly used in document retrieval systems [19]. In [20,21], a 3D shape-based approach was presented by Daras et al. The method relied primarily on the geometric 3D structure of the proteins, which was produced from the corresponding PDB files, and secondarily on their primary and secondary structures. Additionally, characteristic attributes of the primary and secondary structures of the protein molecules were extracted, forming attribute-based descriptor vectors. The descriptor vectors were then weighted and an integrated descriptor vector was produced. To compare a pair of protein descriptor vectors, Daras et al. [20,21] used two metrics of similarity. The first one was based on the *Euclidean distance *[22] between the descriptor vectors, and the second one was based on *Mean Euclidean Distance Measure *[20,21].

Later, Marsolo and Parthasarathy presented two normalized, stand-alone representations of proteins that enabled fast and efficient object retrieval based on sequence or structure information [17,23]. For the range queries, they specified a range value *r *and retrieved all the proteins from the database which lied within a distance *r *to the query. In their work, distance referred to the standard *Euclidean distance *[22]. In [24], Sael et al. introduced a global surface shape representation by 3D Zernike descriptors for protein structure similarity search. In their study, three distance measures were used for comparing 3D Zernike descriptors of protein surface shapes, i.e., *Euclidean distance*, *Manhattan distance *[25], and *correlation coefficient-based distance*. A fast protein comparison algorithm IR Tableau was developed by Zhang et al. for protein retrieval purposes in [26], which leveraged the tableau representation to compare protein tertiary structures. IR tableau compared tableaux using feature indexing techniques. In IR Tableau [26], a number of similarity functions were applied for comparing a pair of protein vectors, i.e., *cosine similarity *[27], *Jaccard index *[28], *Tanimoto coefficient *[29], and *Euclidean distance*.

The basic components of a protein retrieval system includes a way to represent proteins and a dissimilarity measure that compares a pair of proteins. Most of the aforementioned studies focus on the feature representation of the proteins, while neglecting the comparison of the feature vectors. Such studies usually apply a simple similarity or dissimilarity measure for the comparison of the feature vectors, such as Euclidean Distance Measure used in [17,20,21,23,24,26]. Most of the existing protein comparison techniques suffer from the following two bottlenecks:

• The dissimilarity measure is a pairwise distance measure, which is computed only considering the query protein *x*_0 _and a database protein *x_i _*as *d*(*x*_0_, *x_i_*). It does not consider other proteins in the database, neglecting the effects of the contextual proteins. If we consider the distribution of the entire protein database *X *= {*x_j_*}, *j *= 1 ... *N *when computing the dissimilarity as *d*(*x*_0_, *x_i_|X*), the retrieval performance may benefit from the contextual proteins {*x_j_*}, *j *≠ *i*.

• The dissimilarity measure is computed in an unsupervised way, which does not use the known information of the class labels *L *= {*l_j_*}, *j *= 1 ... , *N *in the database. Although we may have no idea about whether *x*_0 _and *x_i _*belong to the same class (having the same folding type etc., *l*_0 _= *l_i_*) or not (*l*_0 _≠ *l_i_*), we do know some prior information about other proteins *L*. In all of the previous studies, prior class labels *L *were not adopted to calculate the dissimilarity *d*(*x*_0_, *x_i_*).

Due to these two bottlenecks, traditional protein retrieval systems using pairwise and unsupervised dissimilarity measure usually do not achieve satisfactory performance, even though many effective protein feature descriptors are developed and used. In this paper, we investigate the dissimilarity measure and propose a novel learning algorithm to improve the performance of a given dissimilarity measure.

Recent research in machine learning points out that contextual information can be used to improve the dissimilarity or similarity measures. This kind of algorithms are called contextual or context-sensitive dissimilarity learning [30-34]. Unlike the traditional pairwise distance *d*(*x*_0_, *x_i_*) which only considers the two refereed proteins *x*_0 _and *x_i_*, contextual dissimilarity also considers the contextual proteins *X *when computing the dissimilarity *d*(*x*_0_, *x_i_|X*). The existing contextual similarity learning algorithms can mainly be classified into the following two categories:

### Dissimilarity regulation

The first contextual dissimilarity measure (CDM) was proposed by Jegou et al. in [30,31]. They introduced the CDM, which significantly improved the accuracy of the image search problem. CDM measure took the local distribution of the vectors into account and iteratively estimated the distance update terms in the spirit of Sinkhorns scaling algorithm [35], thereby modified the neighborhood structure. This regularization was motivated by the observation that a good ranking was usually not symmetric in an image search system. In this paper, we will focus on this type of contextual dissimilarity learning.

### Similarity transduction on graph

In [32,33], Bai et al. provided a novel perspective to the shape retrieval tasks by considering the existing shapes as a group and studying their similarity measures to the query shape in a graph structure. For a given similarity measure, a new similarity was learned through graph transduction. The learning was done in an iterative manner so that the neighbors of a given shape influenced the final similarity to the query. The basic idea is actually related to the PageRank algorithm, which forms a foundation of Google Web search. This method is further improved by Wang et al. in [36]. Similar learning algorithms were also used to rank proteins in a protein database as in [37,38]. Kuang et al. proposed a general graph-based propagation algorithm called MotifProp to detect more subtle similarity relationship than the pairwise comparison methods. In [38], Weston et al. reviewed RankProp, a ranking algorithm that exploited the global network structure of similarity relationship among proteins in a database by performing a diffusion operation on a protein similarity network with weighted edges.

The drawbacks of the above algorithms lay on two folds. On the one hand, such algorithms do not utilize the class label information of the database images *L*, and thus work in an unsupervised way. The only one used *L *is [38]. However, the algorithm proposed in [38] had basically the same framework as [32,33,37], i.e., protein label information *L *was only used to estimate the parameters. On the other hand, the "context" is fixed in the iterative algorithms of most of the transduction methods [32,33,37,38]. A better way is to update the context using the learned similarity measures as in [30,31].

To overcome these drawbacks, we develop a novel contextual dissimilarity learning algorithm to improve the performance of a protein retrieval system. The novel dissimilarity measure is regularized by the dissimilarity of the contextual proteins (neighboring proteins), while the contextual proteins are updated using the learned dissimilarities coherently. The basic idea comes from [39,40], which assume that if two local features in two images are similar, their context is likely to be similar. In comparison to [30,31], which use neighborhood as a single context, we partition the neighborhood into several hierarchical sub-context corresponding to the learned dissimilarities. With the sub-context, we compute the dissimilarity of sub-context of a pair of proteins and construct the hierarchial sub-contextual dissimilarity vector. Moreover, using the label information *L*, we select pairs of proteins belonging to the same classes {(*x_i_*, *x_j_*)*|l_i _*= *l_j_*} as the relevant protein pairs. We also select the irrelevant protein pairs {(*x_k_*, *x_l_*)*|l_k _*≠ *l_l_*}.

Finally, we train a support vector machine (SVM) [41] to distinguish between the relevant and the irrelevant protein pairs. The output of the SVM will further be used to regularize the dissimilarity in an iterative manner.

## Methods

This section describes our contextual protein-protein dissimilarity learning algorithm, which utilizes the contextual proteins and class label information of the database proteins to index and search protein structures efficiently. We will demonstrate that our idea is general in the sense that it can be used to improve the existing similarity/dissimilarity measures.

### Protein structure retrieval framework

**Figure 1:**
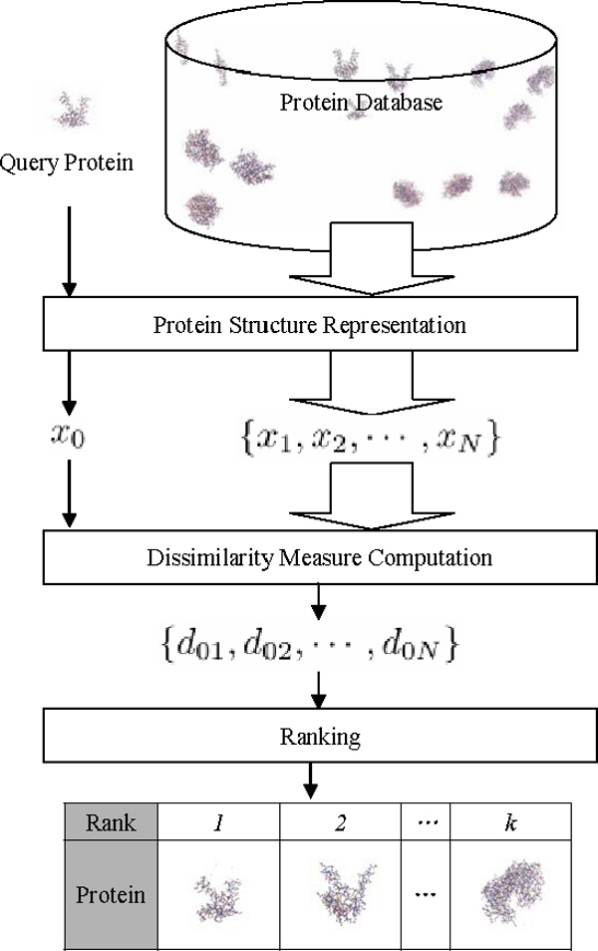
**Flowchart of protein retrieval systems**.

In a protein retrieval system, the query and the database proteins are firstly represented as feature vectors. Here, we denote the query protein feature vector as *x*_0 _and database protein feature vectors as *X *= {*x*_1_, *x*_2_, ... , *x_N_*}, where *N *is the number of proteins in the database. Then, based on a distance measure *d*_0*i*_= *d*(*x*_0_, *x_i_*), we compute the distance of *x*_0 _and all the proteins in the database, i.e., {*d*_01_, *d*_02_, ... , *d*_0*N*_}. The database proteins are then ranked according to the distances. The *k *most similar ones are returned as the retrieval results. We illustrate the outline of the protein retrieval system in Figure 1.

### ProDis-ContSHC: the contextual dissimilarity learning algorithm

In this section, we will introduce the novel contextual protein-protein dissimilarity learning algorithm. We first give the definition of the hierarchical context of a protein, which will be used to compute the contextual dissimilarity and regularize the dissimilarity measure. Then a more discriminative regularization factor is learned using the class labels of the database proteins. Finally, we propose the **S**upervised regulating of **Pro**tein-protein **Dis**similarity and updating of the **H**ierarchical **Cont**ext **C**oherently in an iterative manner, resulting in the ProDis-ContSHC algorithm.

#### Using hierarchical context to regularize the dissimilarity measure

Here, we define a protein *x_i_*'s context as its *K *nearest neighbors N(i). The dissimilarity between two sets of context is measured by the contextual dissimilarity as

(1) $$r_{ij}=\frac{1}{K^{2}}\underset{m\in N\left(i\right),n\in N\left(j\right)}{\sum }d_{mn}$$

**Figure 2:**
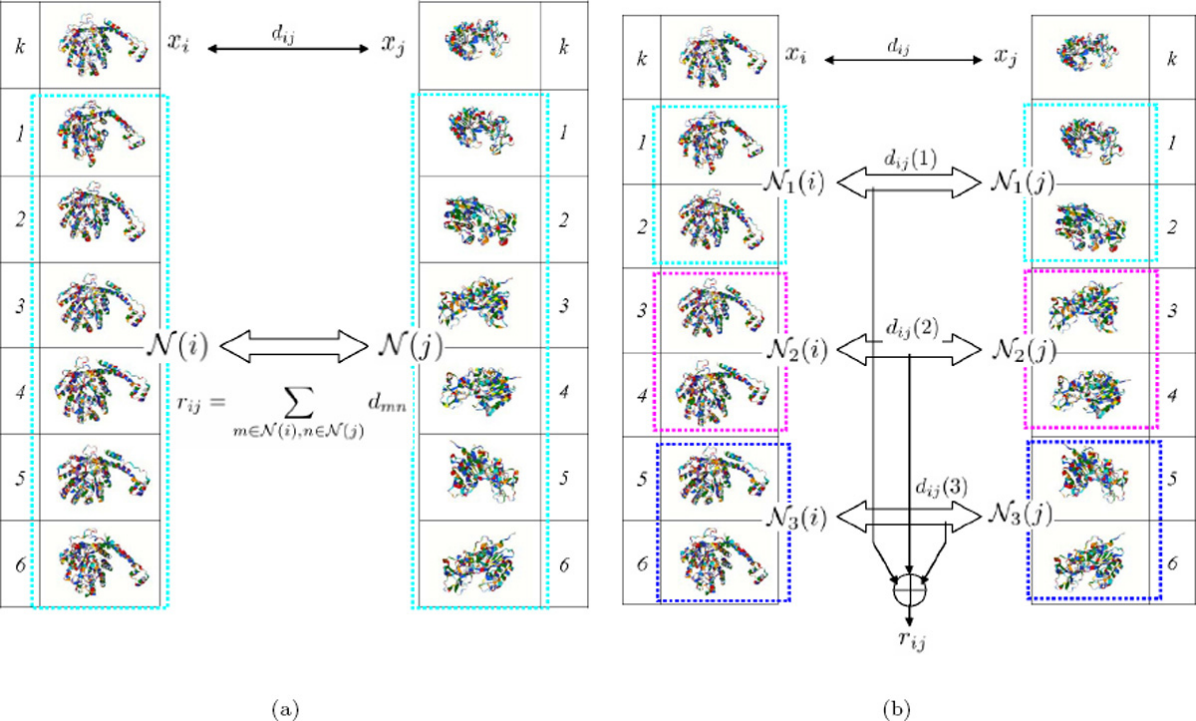
**Illustration of context-based dissimilarity and hierarchical context-based dissimilarity**. The two proteins *x_i _*and *x_j_*, on which the dissimilarity is to be measured, are in the first row. The nearest neighbors of these two proteins are listed below them as the context, respectively. (a) The traditional context N(i); (b) The proposed hierarchical context $N_{p}\left(i\right)$, *p *= {1, 2, 3}.

The contextual dissimilarity is illustrated in Figure 2(a).

Furthermore, instead of averaging all the pairwise dissimilarities between the two context N(i) and N(j), we propose the hierarchical context by splitting the context N(i) to *P *"sub-context" $N_{p}\left(i\right),p=\left\{1,\cdots \;,P\right\}$ according to their distances to *x_i_*. To be more specific, sub-context $N_{p}\left(i\right)$ is defined as

(2) $$\begin{array}{llll} N_{p}\left(i\right) & =\{x_{j}|x_{j}\;is\;among\;the\;k^{\prime }\;-th\;to\;k^{\prime \prime }\;-th\; & & \;\\ & \;nearest\;neighbors\;of\;x_{i},\;according\;to\;\left\{d_{ij}\right\},\; & & \;\\ & \;j\in \left\{1,\;\cdots \;,\;i-1,\;i+1,\;\cdots \;,\;N\right\}\}\; & & \;\\ & \; & \end{array}$$

where *k*' = (*p - *1) *× κ*, *k*'' = (*p - *1) *× κ *+ *κ*, *κ *is the size of a sub-context, and *P *is the number of sub-context. In this way, we can compute the contextual dissimilarity by averaging the dissimilarity of the sub-context as

(3) $$\begin{array}{llll} r_{ij} & =\frac{1}{P}\underset{p}{\sum }\left[\frac{1}{\kappa^{2}}\underset{m\in N_{p}\left(i\right),n\in N_{p}\left(j\right)}{\sum }d_{mn}\right]\; & & \;\\ & =\frac{1}{P}\underset{p}{\sum }d_{ij}\left(p\right)\; & & \;\\ & \; & \end{array}$$

where $d_{ij}\left(p\right)=\frac{1}{\kappa^{2}}\sum_{m\in N_{p}\left(i\right),n\in N_{p}\left(j\right)}d_{mn},p=1,\cdots \;,P$, is the hierarchical sub-contextual dissimilarity. Figure 2(b) illustrates the idea of sub-contextual dissimilarity.

Intuitively, if the context of two proteins is dissimilar to each other (*r_ij _*is higher than the average), they should have a higher dissimilarity value, and vice versa. We implement this by multiplying a coefficient, which is the ratio of *r_ij _*to the average of all the contextual dissimilarity $\bar{r}=\frac{1}{N^{2}}\sum_{i,j}r_{ij}$,

(4) $$\begin{array}{llll} d_{ij}^{*} & =d_{ij}\times \frac{r_{ij}}{\bar{r}}\; & & \;\\ & =d_{ij}\times \delta_{ij}\; & & \;\\ & \; & \end{array}$$

Here, $\delta_{ij}=\frac{r_{ij}}{\bar{r}}$ is a regularization factor for *d_ij_*, with which we can improve *d_ij _*by its contextual information. Moreover, this procedure can be done in an iterative manner. We can use the regularized dissimilarity measure $d_{ij}^{*}$ to re-define the new hierarchical context $N_{p}\left(i\right)$. In this way, we can learn the protein-protein dissimilarity $d_{ij}^{*}$ and hierarchical context $N_{p}\left(i\right)$ coherently.

#### Supervised regularization factor learning

**Figure 3:**
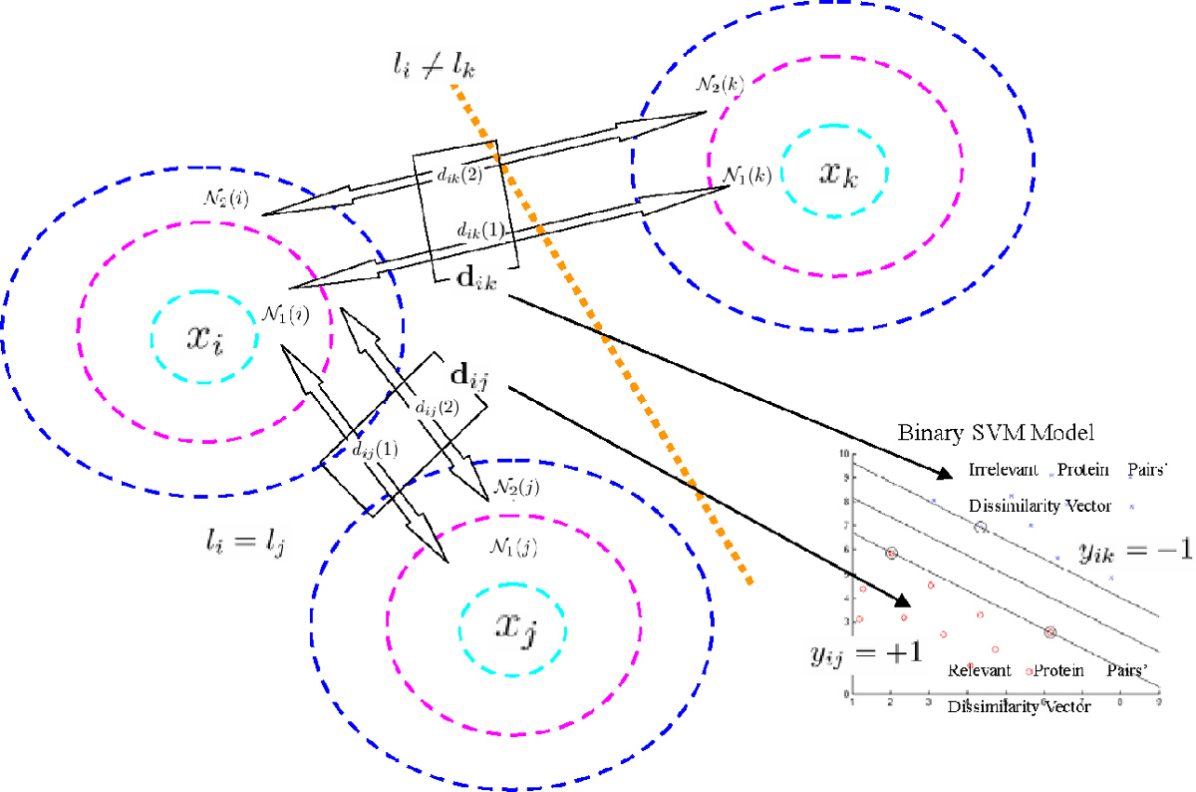
**Differentiate relevant and irrelevant proteins by classification**. (*x_i_*, *x_j_*) is assumed to be a relevant pair and (*x_i_*, *x_k_*) is assumed to be an irrelevant pair. The contextual dissimilarity vectors of both pairs are distinguished by a binary SVM model.

We try to utilize the label information *L *= {*l*_1_, ... , *l_N_*} of the database proteins to learn a better regularization factor *δ_ij_*. The class information is adopted both in the intraclass and interclass dissimilarity computation to maximize the Fisher criterion [42] for protein class separability. Firstly, we can select a number of protein pairs {*γ *= (*i*, *j*)*|i*, *j *= 1, ... , *N*}. For each pair, we compute the hierarchical contextual dissimilarities and organize them as a *P*-dimensional dissimilarity vector **d***_γ _*= [*d_ij _*(1) *d_ij _*(2) ... *d_ij _*(*P*)]^⊤^, as shown in Figure 3. Then, inspired by the score fusion rule [43,44], using *L*, we further label each pair *γ *= (*i*, *j*) as a relevant pair *y_γ _*= +1 if *l_i _*= *l_j_*, or an irrelevant pair *y_γ _*= *-*1 otherwise.

Now with the training samples as Γ = {(**d***_γ_*, *y_γ_*)}, *γ *= 1, ... ,*_N _C*_2_, we train a binary SVM [41] classifier to distinguish between the relevant pairs and the irrelevant pairs. The publicly available package SVMlight [45] is applied to implement the SVM on our training set Γ. This package allows us to optimize a number of parameters and offers the options to use different kernel functions to obtain the best classification performance [46]. The separating hyperplane generated by SVM model is given by

(5) f(d)=d⋅w+b

where *w *is a vector orthogonal to the hyperplane, and *b *is a parameter that minimizes ||*w*||^2 ^and satisfies the following conditions:

(6) $$y_{\gamma }\left(d_{\gamma }\cdot w+b\right)\geq 1$$

for all 1 *≤ γ ≤ _N _C*_2_, where *_N_C*_2 _is the total number of examples (protein pairs). An SVM model with a linear decision boundary is shown in Figure 3 to distinguish the relevant protein pairs from the irrelevant ones. Note that not all the *_N_C*_2 _possible protein pairs are necessary to be included to train the SVM model (5). For any pair of proteins (*x_i_*, *x_j_*), after we compute its contextual dissimilarity vector **d***_ij_*, the trained SVM classifier is applied to get the distance of this point to the margin boundary of SVM as ${ỹ}_{ij}=f\left(d_{ij}\right)$. Apparently, ${ỹ}_{ij}$ is a measure of dissimilarity of the context of this pair of proteins. Thus, it can be used to form a regularization factor as

(7) $$\begin{array}{llll} {\delta^{\prime }}_{ij} & =exp\left(-\frac{{\tilde{y}}_{ij}}{\sigma }\right)\; & & \;\\ & =exp\left[-\frac{\left(d_{ij}\cdot w+b\right)}{\sigma }\right]\; & & \;\\ & \; & \end{array}$$

where *σ *is a preemptor of the factor. With this regularization factor learned from the contextual proteins, we regularize the dissimilarity *d_ij _*of protein pair (*x_i_*, *x_j_*) as

(8) $$d_{ij}^{*}=d_{ij}\times \delta_{ij}^{\prime }$$

#### Updating the context and dissimilarity coherently

With the learned dissimilarity measure $d_{ij}^{*}$, we can re-define the "context" of a protein *x_i _*according to its dissimilarity to all the other proteins $d_{ij}^{*},j\in \left\{0,\;\cdots \;,\;i-1,\;i+1,\;\cdots \;,\;N\right\}$. The new "hierarchical-context" relying on $d_{ij}^{*}$ is donated as $N_{p}^{*}\left(i\right),p=\left\{1,\;\cdots \;,\;P\right\}$. In this way, we can develop an iterative algorithm that learns $d_{ij}^{*}$ and $N_{p}^{*}\left(i\right),p=\left\{1,\;\cdots \;,\;P\right\}$ coherently. Since $N_{p}^{*}\left(i\right)$ implicitly depends on $d_{ij}^{*}$ through the nearest neighbors of *x_i_*, we use a fixed-point recursion method [47] to solve $d_{ij}^{*}$. In each iteration, $N_{p}^{*}\left(i\right)$ is first computed by using the previous estimation of $d_{ij}^{*}$, which is then updated by multiplying the regularization factor $\delta_{ij}^{\prime }$ as in (8). The iterations are carried out for *T *times, as given in Algorithm 1.

With the learned dissimilarity matrix **D**^(*t*+1)^, we use **D**^(*t*+1)^[0; 1, ... , *N*] as the dissimilarity between the query protein *x*_0 _and the database proteins {*x*_1_, ... , *x_N_*}. Thus we can rank the database proteins in an ascending order.

#### Efficient implementation of ProDis-ContSHC

The proposed learning algorithm is time-consuming. Therefore, it is not suitable for realtime protein retrieval systems. Here we propose several techniques to significantly improve the efficiency of the algorithm.

**Figure 4:**
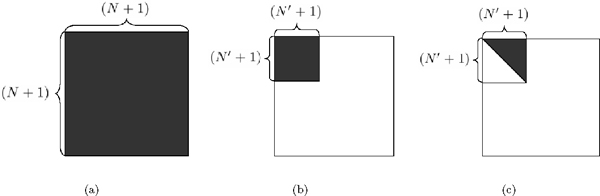
**Efficient implementation of ProDis-ContSHC**. (a) Performing ProDis-ContSHC on the original matrix of size (*N *+ 1) *× *(*N *+ 1) from the entire dataset; (b) Performing ProDis-ContSHC on a subset of the database proteins, i.e., a dissimilarity matrix of size (*N*' + 1) *× *(*N*' + 1); (c) Using the symmetry property of the dissimilarity matrix to reduce the training time.

• Similar to [33], in order to increase the computational efficiency, it is possible to run ProDis-ContSHC for only part of the database of the known proteins. Hence, for each query protein *x*_0_, we first retrieve *N*' ≪ *N *of the most similar proteins, and perform ProDis-ContSHC to learn the dissimilarity matrix of size (*N*' + 1) *× *(*N*' + 1) for only those proteins. Then we calculate the new dissimilarity measure *D*' _(*N*' + 1) × (*N*' + 1) _for only those (*N*' + 1) proteins. Here, we assume that all the relevant proteins will be among the top *N*' most similar proteins. This strategy is illustrated in Figure 4(a) and 4(b).

• Most of the dissimilarity and similarity measures are symmetric ones, i.e., *d_ij _*= *d_ji_*. As can be observed in (13), the regularization of *d_ij _*is also symmetric. Therefore, it is possible to develop an efficient learning algorithm by using this property. In the algorithm, all the computation results of (*i*, *j*) (such as **d***_ij _*and *δ_ij_*) can be used directly by (*j*, *i*). In this way, we can save almost half of the computational time, as shown in Figure 4(c).

• A bottleneck of ProDis-ContSHC may be the training procedure for the SVM model in each iteration. For a database of *N *proteins belonging to *C *classes, there are *_N _C*_2 _protein pairs, in which $\sum_{c=1}^{C}{}_{N_{c}}C_{2}$ are relevant pairs, while $\sum_{c=1}^{C}\sum_{c^{\prime }\neq c}N_{c}\times N_{c^{\prime }}$ are irrelevant pairs, where *C *is the number *C *of the protein classes and *N_c _*is the number of proteins in the *c*-th class $\left(\sum_{c=1}^{C}N_{c}=N\right)$. There might be a huge number of protein pairs available for the SVM training. However, it is not necessary to include all of them in the training process. One can select a small but equal number of the relevant and the irrelevant pairs to train the SVM classifier. This is an effective way to reduce the training time of SVM.

**Algorithm 1 **ProDis-ContSHC: **S**upervised Learning of **Pro**tein **Dis**similarity and Updating **H**ierarchical **Cont**ext **C**oherently.

**Require: **Input **D **= [*d_ij_*]_(*N*+1)*×*(*N*+1)_: matrix of size (*N*+1)×(*N*+1) of pairwise protein feature distances, where *x*_0 _is the query protein and {*x*_1_, ... , *x_N_*} are the database proteins;

**Require: **Input *κ*: size of the hierarchical sub-context;

**Require: **Input *P*: number of the hierarchical context;

Initialize dissimilarity matrix: **D**^(1) ^= **D**;

**for ***t *= 1, ... , *T ***do**

Update the hierarchical context for each protein $x_{i}:N_{p}^{\left(t\right)}\left(i\right),\left(p=1,\;\cdots \;,\;P\right)$,

(9) $$\begin{array}{llll} N_{p}^{\left(t\right)}\left(i\right) & =\{x_{j}|x_{j}\;is\;among\;the\;k^{\prime }\;-th\;to\;k^{\prime \prime }\;-th\; & & \;\\ & \;\;nearest\;neighbors\;of\;x_{i},\;according\;to\; & & \;\\ & D^{\left(t\right)}\left(i;1,\;\cdots \;,\;N\right)\}\; & & \;\\ & \; & \end{array}$$

where *k*' = (*p - *1) *× κ*, *k*'' = (*p - *1) *× κ *+ *κ*, and $D^{\left(t\right)}\left(i;0,\;\cdots \;,\;N\right)=\left[d_{i0}^{\left(t\right)},\;\cdots \;,\;d_{iN}^{\left(t\right)}\right]$.

Compute the contextual proteins dissimilarity vector $d_{ij}^{\left(t\right)}$ for each pair of proteins (*i*, *j*), *i*, *j ∈ *{0, ... , *N*}:

(10) $$d_{ij}^{\left(t\right)}={\left[d_{ij}^{\left(t\right)}\left(1\right)\;d_{ij}^{\left(t\right)}\left(2\right)\;\cdots \;d_{ij}^{\left(t\right)}\left(P\right)\right]}^{⊤}$$

where $d_{ij}^{\left(t\right)}\left(p\right)=\frac{1}{k^{2}}\sum_{m\in N_{p}^{\left(t\right)}\left(i\right),n\in N_{p}^{\left(t\right)}\left(j\right)}d_{mn}^{\left(t\right)}$.

Select relevant and irrelevant protein pairs and label them as *y_γ _*= +1 and *y_γ _*= *-*1 respectively, train an SVM model for their contextual dissimilarity vectors $d_{\gamma }^{\left(t\right)}$ as

(11) $$f^{\left(t\right)}\left(d\right)=w^{\left(t\right)}\cdot d+b^{\left(t\right)}$$

Compute the distance to the SVM margin boundary for the contextual dissimilarity vector $d_{ij}^{\left(t\right)}$ of each pair of proteins as ${ỹ}_{ij}^{\left(t\right)}=f^{\left(t\right)}\left(d_{ij}^{\left(t\right)}\right)$, and set a regularization factor for this pair of proteins:

(12) $$\delta_{ij}^{\left(t\right)}=exp\left(-\frac{{\tilde{y}}_{ij}^{\left(t\right)}}{\sigma }\right)$$

Update the pairwise protein dissimilarity measures:

**for ***i *= 0, 1, ... , *N ***do**

**for ***j *= 0, 1, ... , *N ***do**

(13) $$d_{ij}^{\left(t+1\right)}=d_{ij}^{\left(t\right)}\times \delta_{ij}^{\left(t\right)}$$

end for

end for

**$D^{\left(t+1\right)}={\left[d_{ij}^{\left(t+1\right)}\right]}_{\left(N+1\right)\times \left(N+1\right)}$**.

end for

Output the dissimilarity matrix: **D**^(*t*+1)^.

### Benchmark sets

To evaluate the proposed ProDis-ContSHC algorithm, we conduct experiments on two different benchmark sets, i.e., the ones used in [21] and [26] respectively.

#### ASTRAL 1.73 protein domain dataset

Following [26], we use the following database and queries as our first benchmark set:

##### Database

The ASTRAL 1.73 [48] 95% sequence-identity non-redundant data set is used as the protein database. We generate our index database from the tableau data set published by Stivala et al. [49], which contains 15,169 entries.

##### Queries

A query data set containing 200 randomly selected protein domains is used in our experiment. For each query, a list that contains all the proteins in the respective index database is returned with the ranking scores.

We generate a vector of features *x *for a given protein based on its tableau representation [49].

#### FSSP/DALI protein dataset

To evaluate the performance of the proposed methods, a portion of the FSSP database [50] is selected as in [21]. This dataset has 3,736 proteins classified into 30 classes. It’s constructed according to the DALI algorithm [51,52]. The protein numbers in different classes varies 2 to 561. For protein feature representation, the following two features are extracted from the 3D structure and the sequence of a protein as in [20,21]:

• The Polar-Fourier transform, resulting in the *FT*_02 _features;

• Krawtchouk moments, resulting in the *Kraw*_00 _features.

The descriptor vectors are weighted and an integrated descriptor vector is produced as *x*, which will be used for the protein retrieval tasks.

## Results and discussion

### Results on ASTRAL 1.73 dataset

To compare a query protein *x*_0 _to a protein *x_i _*in the ASTRAL 1.73 dataset, we compute the cosine similarity [27] as the baseline similarity measure as in [26]. Cosine similarity [27] simply calculates the cosine of the angle between the two vectors *x_i _*and *x_j_*.

(14) $$s_{ij}=C\left(x_{i},\;x_{j}\right)=\frac{x_{i}\cdot x_{j}}{\left||x_{i}||\;||x_{j}|\right|}$$

A higher cosine similarity score implies a smaller angle between the two vectors. Although ProDis-ContSHC is proposed to learn protein-protein dissimilarity *d_ij_*, it can be extended easily to learn similarity *s_ij _*as well. The only difference is to set the regularization factor as $\delta_{ij}^{\prime }=exp\left(\frac{{\tilde{y}}_{ij}}{\sigma }\right)$ instead of $\delta_{ij}^{\prime }=exp\left(-\frac{{\tilde{y}}_{ij}}{\sigma }\right)$ in (7).

#### ROC curve and precision-recall curve performance

SCOP [53] fold classification is used as the ground truth to evaluate the performance of the different methods. To fairly compare the accuracy, we use the receiver operating characteristic (ROC) curve [54], the area under this ROC curve (AUC) [54], and the precision-recall curve [55]. Given a query protein *x*_0 _which belongs to the SCOP fold *l*_0_, the top *k *proteins returned by the search algorithms are considered as the *hits*. The remaining proteins are considered as the *misses*. For the *i*-th protein *x_i _*belonging to the SCOP fold *l_i_*, if *l_i _*= *l*_0 _and *i *≤ *k*, the protein *x_i _*is defined as a true positive (TP). On the other hand, if *l_i _*≠ *l*_0 _and *i ≤ k*, *x_i _*is defined as a false positive (FP). If *l_i _*≠ *l*_0 _and *i > k*, *x_i _*is defined as a true negative (TN). Otherwise, *x_i _*is a false negative (FN). Using these definitions, we can then compute the true positive rate (TPR or recall), the false positive rate (FPR), recall and precision as follows:

(15) $$\begin{array}{c} TPR=\frac{TP}{P}=\frac{TP}{TP+FN}\\ FPR=\frac{FP}{N}=\frac{FP}{FP+TN} \end{array}$$

(16) $$\begin{array}{c} Recall\;=\frac{TP}{TP+FN}\\ Precision\;=\frac{TP}{TP+FP} \end{array}$$

*TPR_k_*, *FPR_k_*, *Recall_k_*, and *Precision_k _*are calculated for all 1 ≤ *k *≤ *N *, where *N *is the size of the database. The ROC defines a curve of points with *FPR_k _*as the abscissa and *TPR_k _*as the ordinate. Precision-recall defines a curve with *recall_k _*and *precision_k _*as abscissa and ordinate respectively. We use the area under the ROC curve (AUC) as a single-figure measurement for the quality of a ROC curve [54], and use the averaged AUC over all the queries to evaluate the performance of the method.

To demonstrate the contribution of the supervised learning idea, we also compare ProDis-ContSHC with its unsupervised counterpart, i.e., contextual dissimilarity algorithm based on the unsupervised learning, i.e., ProDis-ContHC. ProDis-ContHC is also applied to improve the cosine similarity. We also compare with the widely-used contextual dissimilarity measure [30,31] (CDM), which tries to take into account the local distribution of the vectors and iteratively estimates distance update terms in the spirit of Sinkhorns scaling algorithm, thereby modifying the neighborhood structures.

**Figure 5:**
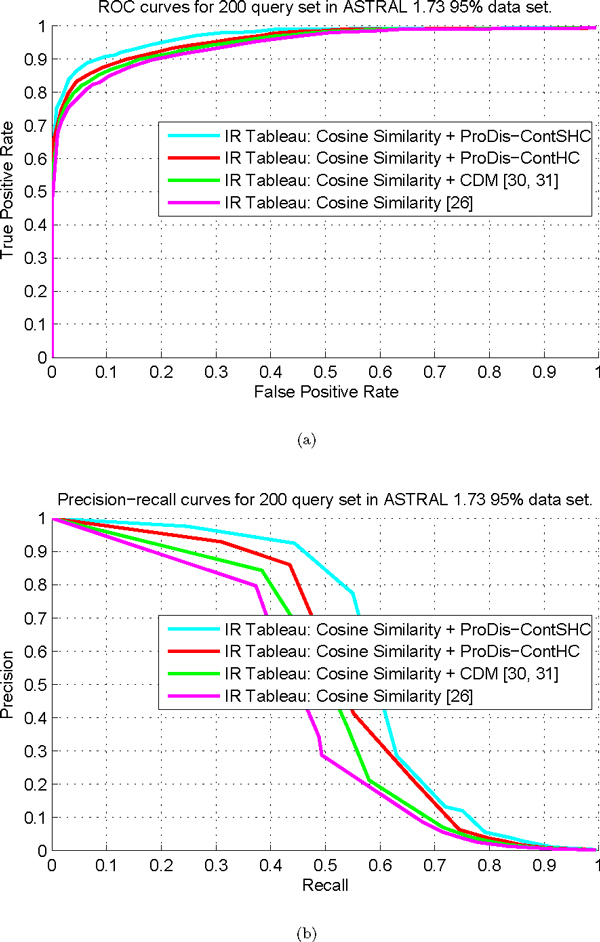
**Performance of similarity measures on the ASTRAL 1.73 90% dataset**. (a) The ROC curves of the original similarity measure, and the improved measures by ProDis-ContSHC, ProDis-ContHC, and CDM, respectively. (b) The precision-recall curves of the original similarity measure, and the improved measures by ProDis-ContSHC, ProDis-ContHC, and CDM, respectively.

The performance of different methods are compared, as shown in Figure 5. Figure 5(a) shows the ROC curves of the original cosine similarity and its improved versions by three contextual similarity learning algorithms on the ASTRAL 1.73 [48] 95% dataset, with different numbers of proteins returned to each query. It can be seen from Figure 5(a) that the TPR of all the methods increases as the FPR grows. The reason is due to the fact that, provided the number of queries is fixed, when the number *k *of returned proteins to each query is very small, the returned proteins are not enough to "represent" the class features of the query, which then causes the low TPR. Meanwhile, in this situation, most of the returned proteins are highly confident of belonging to the same class as the query, resulting in a low FPR. Moreover, the TPR is almost 100% when the FPR*>*50%. It is clear that the ROC curve of ProDis-ContSHC completely embodies the ROC curves of the other three methods, which implies ProDis-ContSHC is the best method among the four. That also means that supervised learning is better than unsupervised learning for this purpose. ProDis-ContHC, on the other hand, is the second best method among these four, which demonstrates the contribution of the hierarchical sub-context idea to the traditional contextual dissimilarity measures. The overall AUC results are listed in Table 1, from which similar conclusions can be drawn. It is noticeable that the AUC for ProDis-ContSHC is very close to 1, which means ProDis-ContSHC works almost perfectly on this dataset. We further compare these four methods by the precision-recall curves, which are shown in Figure 5(b). It can be seen that the proposed contextual similarity learning algorithms significantly outperform the traditional methods. ProDis-ContSHC, again, is consistently the best method among the four.

**Table 1:** Performance of different retrieval methods on the ASTRAL 1

Method	AUC
IR Tableau: Cosine Similarity + ProDis-ContSHC	0.973
IR Tableau: Cosine Similarity + ProDis-ContHC	0.961
IR Tableau: Cosine Similarity + CDM [30,31]	0.954
IR Tableau: Cosine Similarity [26]	0.948
Tableau Search [56]	0.871
QP Tableau [49]	0.925
Yakusa [57]	0.950
SHEBA [58]	0.941
VAST [59,60]	0.890
TOPS [61,62]	0.871

AUC results for QP Tableau [49], SHEBA [58] and VAST [59,60] are taken from [49], which used exactly the same query set and the same dataset as our experiments.

Regarding the efficiency of the method, in this experiment, the learning time of the ProDis-ContSHC is longer than that of the ProDis-ContHC and CDM. This is because in each iteration of the learning algorithm, a quadratic programming problem with many training protein pairs have to be solved to train the SVM. In addition, the computation of the regularization factor of supervised similarity learning algorithm needs more function evaluations.

We also compare the proposed algorithms with seven other protein retrieval methods, i.e., tableau search [56], QP tableau [49], Yakusa [57], SHEBA [58], VAST [59,60], and TOPS [61,62]. The overall AUC values are shown in Table 1. It can be concluded that the tableau feature based methods do not always achieve better performance than other methods, such as tableau search. Among the existing tableau feature based methods, IR tableau outperforms the others. Yakusa and SHEBA also have comparable performance. As seen in Table 1, the AUC of the proposed algorithms is clearly better than all the other methods.

#### Improving different similarity measures via contextual dissimilarity learning algorithms

To further evaluate the robustness of our method, we test the behavior of ProDis-ContSHC and other contextual similarity learning algorithms on different similarity measures. A group of experiments are conducted on the ASTRAL 1.73 95% dataset with the following similarity measures:

• The cosine similarity [27] as introduced in the previous section.

• The Jaccard index [28]: it is defined as the size of the intersection divided by the size of the union of two sets, i.e.,

(17) $$J\left(x_{i},\;x_{j}\right)=\frac{\left|x_{i}⋂x_{j}\right|}{\left|x_{i}⋃x_{j}\right|}$$

• The Tanimoto coefficient [29]: it is a generalization of the Jaccard index, defined as

(18) $$J\left(x_{i},\;x_{j}\right)=\frac{x_{i}\cdot x_{j}}{||x_{i}|{|}^{2}+||x_{j}|{|}^{2}-\;x_{i}\cdot x_{j}}$$

• Squared Euclidean distance [22]: it is another means of measuring similarity of proteins.

(19) $$d_{ij}=\sqrt{{\left(x_{i}-x_{j}\right)}^{⊤}(x_{i}-x_{j})}=\sqrt{\sum_{m}{(x_{i}(m)-x_{j}\left(m)\right)}^{2}}$$

where *x_i_*(*m*) is the *m*-th element of vector *x_i_*.

**Figure 6:**
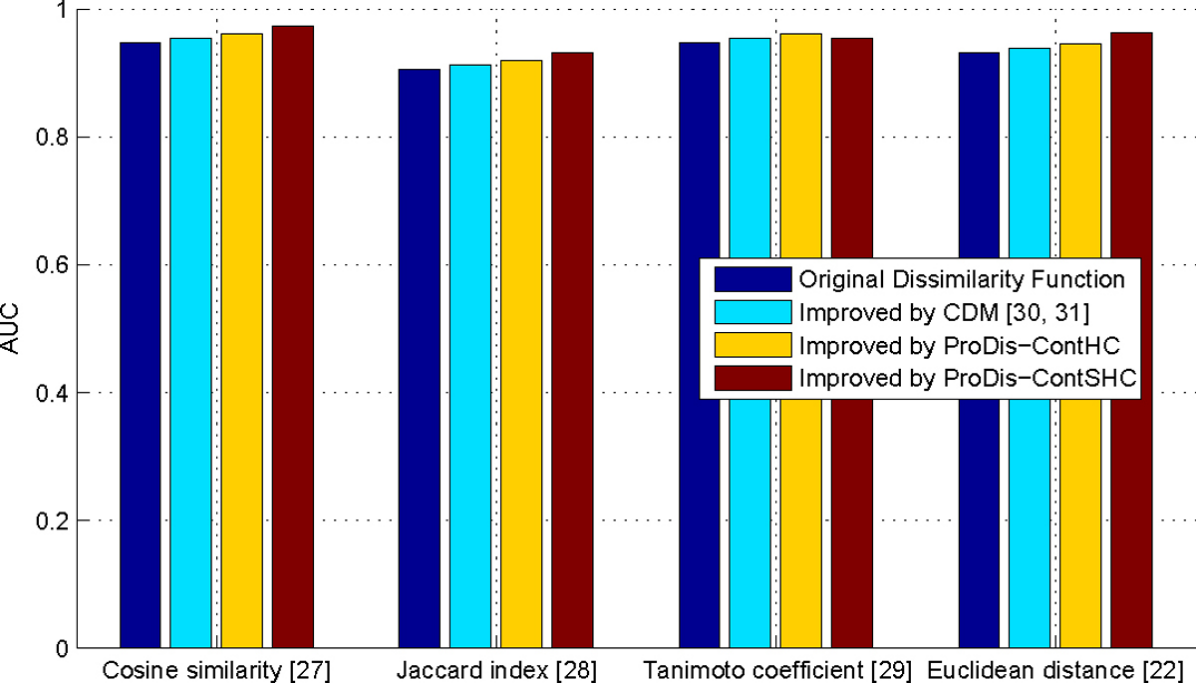
**Performance of similarity measures on different base measures on the ASTRAL 1.73 90% dataset**. Performance of similarity measures on different base measures on the ASTRAL 1.73 90% dataset. The four base measures being tested are cosine similarity [27], the Jaccard index [28], the Tanimoto coefficient [29], and the Euclidean distance [22].

ProDis-ContSHC, ProDis-ContHC, and the CDM algorithms are applied to improve each of these similarity measures, respectively. The AUC values of the corresponding retrieval systems are plotted in Figure 6. In general, improving the original similarity measure by ProDis-ContSHC leads to the largest improvement. The only exception is for Tanimoto coefficient, on which ProDis-ContSHC has slightly lower AUC than ProDis-ContHC, but comparable AUC to the CDM. One possible reason is that the supervised classifier fail to capture the real distribution of the contextual similarity. ProDis-ContHC, on the other hand, also performs better than the CDM algorithm and the original similarity measures. This strongly suggests that our previous conclusions are valid and consistent. That is, hierarchical sub-contextual information can remarkably improve the traditional context-based similarity measures, whereas supervised learning can further improve the accuracy for most of the input similarity measures.

### Results on FSSP/DALI dataset

Unlike the similarity measure used in the last experiment, here we use the Euclidean distance [22] to compare a pair of proteins as the baseline dissimilarity measure as in [20,21]. In this way, we have an idea about how our algorithms work with both similarity and dissimilarity measures. For a query protein *x*_0_, the pairwise Euclidean distances, *d*_0*i*_, *i *= 1, 2, ... , *N *, are ranked. The top *k *proteins are returned as the retrieval results. To evaluate the performance of the proposed algorithms, we test them on both the protein retrieval and the protein classification tasks, following [20,21].

#### Performance on protein retrieval

The efficiency of the proposed dissimilarity learning algorithm is first evaluated in terms of the performance on the protein retrieval task. In this case, each protein *x_i _*∈ *X *of the dataset is used as a query *x*_0 _and the retrieved proteins are ranked according to the shape dissimilarity *d*_0*j*_to the query, where *j *= 1, 2, ... , *i *- 1, *i *+ 1, ... , *N*. We also use the precision-recall curve to demonstrate the performance of the proposed methods, where precision is the proportion of the retrieved proteins that are relevant to the query and recall is the proportion of the relevant proteins in the entire dataset that are retrieved as the results.

**Figure 7:**
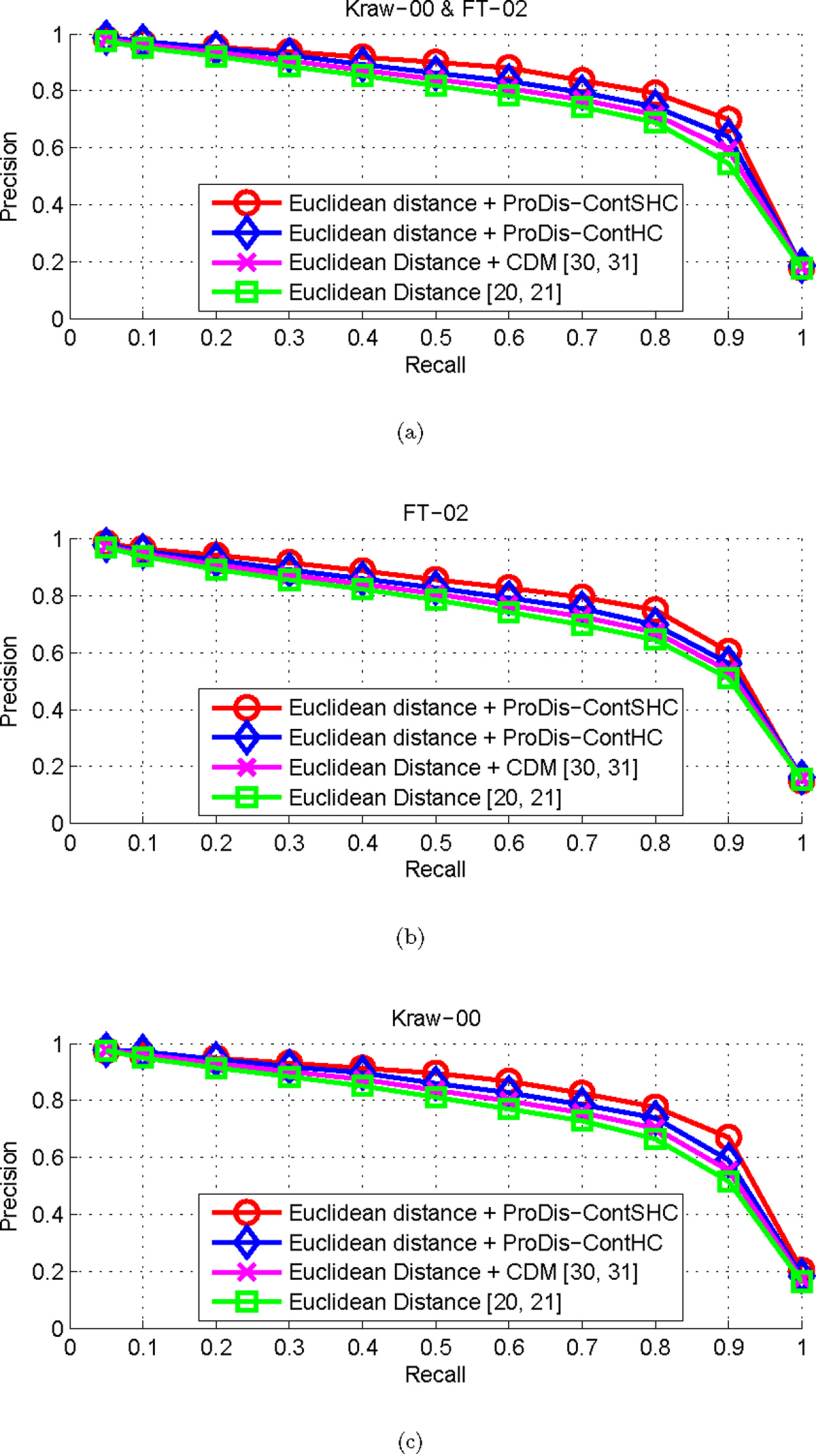
**Performance of dissimilarity measures on the FSSP/DALI dataset**. (a) The precision-recall curves of the original dissimilarity measure, and the improved measures by ProDis-ContSHC, ProDis-ContHC, and CDM, respectively, with the descriptor vector *FT*_02_&*Kraw*_00_. (b) The precision-recall curves with the descriptor vector *FT*_02_. (c) The precision-recall curves with the descriptor vector *Kraw*_00_.

To test the robustness and consistency of our methods, we apply our methods to three different protein descriptor vectors, i.e., Daras et al.'s *FT*_02_, *Kraw*_00_, and *FT*_02_&*Kraw*_00 _[20,21] geometric descriptor vectors. We also apply the unsupervised version of our algorithm, ProDis-ContHC, and the CDM algorithm to the same dissimilarity measure and the same descriptor vectors to compare with ProDis-ContSHC. Figure 7 shows the precision-recall curves for different algorithms on different protein descriptor vectors. As mentioned in [20,21], there is always a tradeoff between the precision and recall values. This is clearly shown in Figure 7(a), (b), and 7(c), in which the algorithms reach their peak precision values at the smallest recall values. It can be seen that ProDis-ContSHC has a clearly better performance than any other method, whereas ProDis-ContHC is the second best one. This is quite consistent with what is observed in the last experiment, in which a similarity measure is used. Therefore, our algorithms can consistently improve any similarity/dissimilarity measure. Among the three protein descriptor vectors, ProDis-ContSHC performs the best on the combined vector, i.e., *Kraw*_00 _&*FT*_02_. This is because this vector not only employs the context, but also their relevant information to predict the relationship between the query and the database proteins.

#### Performance on protein classification

The performance of the method is also evaluated in terms of the overall classification accuracy [20,21]. To be more specific, for each protein *x_i _*in the database, a dissimilarity measure is applied after removing that protein from the database ("leave-one-out" experiment [63]). A class label *l*_0 _is then assigned to the query *x*_0 _according to the label of the nearest database protein. The overall classification accuracy is given by:

(20) $$Overall\;Classification\;Accuracy=\frac{Number\;of\;correctly\;predicted\;proteins}{Total\;number\;of\;proteins\;in\;the\;database}$$

We again conduct this experiment with the three descriptor vectors, i.e., *FT*_02_, *Kraw*_00_, and *FT*_02_&*Kraw*_00_. The overall classification accuracy is shown in Table 2. It can be seen that ProDis-ContSHC has a consistently higher than 99% accuracy on all the three descriptor vectors. Each dissimilarity measure achieves its highest accuracy on *Kraw*_00 _&*FT*_02_. Among the four dissimilarity measures, ProDis-ContSHC has the highest accuracy, whereas ProDis-ContHC is the second best one. Therefore, this conclusion has been demonstrated on both similarity and dissimilarity measures on different datasets with different descriptor vectors.

**Table 2:** Overall classification accuracy using different protein descriptors and the Euclidean distance measure

Dissimilarity measure	Descriptors		
	
	*FT*_02_	*Kraw*_00_	*Kraw*_00 _&*FT*_02_
Euclidean Distance + ProDis-ContSHC	0.9925	0.9954	0.9971
Euclidean Distance + ProDis-ContHC	0.9890	0.9917	0.9928
Euclidean Distance + CDM [30,31]	0.9869	0.9895	0.9909
Euclidean Distance [20,21]	0.9850	0.9879	0.9890

## Conclusions

We have introduced in this paper a novel contextual dissimilarity learning algorithm for protein-protein comparison in protein database retrieval tasks. Its strength resides in the use of the hierarchical context between a pair of proteins and their class label information. By extensive experiments, this novel algorithm has been demonstrated to outperform the traditional context-based methods and their unsupervised version.

We formulate the protein dissimilarity learning problem as a context-based classification problem. Under such a formulation, we try to regularize the protein pairwise dissimilarity in a supervised way rather than the traditional unsupervised way. To the best of our knowledge, this is the first study on supervised contextual dissimilarity learning. We propose a novel algorithm, ProDis-ContSHC, which updates a protein's hierarchical sub-context and the dissimilarity measure coherently. The regularization factors are learned based on the classification of the relevant and the irrelevant protein pairs. The algorithm works in an iterative manner.

Experimental results demonstrate that supervised methods are almost always better than their unsupervised counterparts on all the databases with all the feature vectors. The proposed method, even though mainly presented for protein database retrieval tasks, can be easily extended to other tasks, such as RNA sequence-structure pattern indexing [64], retrieval of high throughput phenotype data [65], and retrieval of genomic annotation from large genomic position datasets [66]. The approach may also be extended to the database retrieval and pattern classification problems in other domains, such as medical image retrieval [67-69], speech recognition, and texture classification [70].

## Competing interests

The authors declare that they have no competing interests.

## Authors' contributions

JW: designed the algorithm, carried out the experiments, analyzed the results, and wrote the manuscript. XG: designed the algorithm and the experiments, improved the manuscript. QW: carried out the experiments, analyzed the results, improved the manuscript. YL: improved the manuscript. All authors read and approved the final manuscript.
